# Capsid Structure of *Leishmania* RNA Virus 1

**DOI:** 10.1128/JVI.01957-20

**Published:** 2021-01-13

**Authors:** Michaela Procházková, Tibor Füzik, Danyil Grybchuk, Francesco Luca Falginella, Lucie Podešvová, Vyacheslav Yurchenko, Robert Vácha, Pavel Plevka

**Affiliations:** a Central European Institute of Technology, Masaryk University, Brno, Czech Republic; b National Centre for Biomolecular Research, Faculty of Science, Masaryk University, Brno, Czech Republic; c Department of Condensed Matter Physics, Faculty of Science, Masaryk University, Brno, Czech Republic; d Life Science Research Centre, Faculty of Science, University of Ostrava, Ostrava, Czech Republic; e Martsinovsky Institute of Medical Parasitology, Tropical and Vector Borne Diseases, Sechenov University, Moscow, Russia; University of Kentucky College of Medicine

**Keywords:** virus, *Leishmania*, *Viannia*, leishmaniasis, parasite, RNA, LRV1, *Totiviridae*, virion, structure, cryo-electron microscopy, capsid, genome, uncoating, mRNA, CAP-4, decapping

## Abstract

*Leishmania* parasites cause a variety of symptoms, including mucocutaneous leishmaniasis, which results in the destruction of the mucous membranes of the nose, mouth, and throat. The species of *Leishmania* carrying *Leishmania* RNA virus 1 (LRV1), from the family *Totiviridae*, are more likely to cause severe disease and are less sensitive to treatment than those that do not contain the virus. Although the importance of LRV1 for the severity of leishmaniasis was discovered a long time ago, the structure of the virus remained unknown. Here, we present a cryo-electron microscopy reconstruction of the virus-like particle of LRV1 determined to a resolution of 3.65 Å. The capsid has icosahedral symmetry and is formed by 120 copies of a capsid protein assembled in asymmetric dimers. RNA genomes of viruses from the family *Totiviridae* are synthetized, but not capped at the 5′ end, by virus RNA polymerases. To protect viral RNAs from degradation, capsid proteins of the L-A totivirus cleave the 5′ caps of host mRNAs, creating decoys to overload the cellular RNA quality control system. Capsid proteins of LRV1 form positively charged clefts, which may be the cleavage sites for the 5′ cap of *Leishmania* mRNAs. The putative RNA binding site of LRV1 is distinct from that of the related L-A virus. The structure of the LRV1 capsid enables the rational design of compounds targeting the putative decapping site. Such inhibitors may be developed into a treatment for mucocutaneous leishmaniasis caused by LRV1-positive species of *Leishmania*.

**IMPORTANCE** Twelve million people worldwide suffer from leishmaniasis, resulting in more than 30 thousand deaths annually. The disease has several variants that differ in their symptoms. The mucocutaneous form, which leads to disintegration of the nasal septum, lips, and palate, is caused predominantly by *Leishmania* parasites carrying *Leishmania* RNA virus 1 (LRV1). Here, we present the structure of the LRV1 capsid determined using cryo-electron microscopy. Capsid proteins of a related totivirus, L-A virus, protect viral RNAs from degradation by cleaving the 5′ caps of host mRNAs. Capsid proteins of LRV1 may have the same function. We show that the LRV1 capsid contains positively charged clefts that may be sites for the cleavage of mRNAs of *Leishmania* cells. The structure of the LRV1 capsid enables the rational design of compounds targeting the putative mRNA cleavage site. Such inhibitors may be used as treatments for mucocutaneous leishmaniasis.

## Abstract (precis)

Twelve million people worldwide suffer from leishmaniasis, resulting in more than 30 thousand deaths annually. The disease has several variants that differ in their symptoms.

## INTRODUCTION

Parasites from the genus *Leishmania* (Kinetoplastea: Trypanosomatidae) cause a range of diseases, known as leishmaniases, which affect 12 million people worldwide and result in more than 30 thousand deaths annually (1, 2). *Leishmania* parasites are transmitted to their mammalian hosts, including humans, by the bite of an infected sand fly or a midge (3). The disease can develop into three variants that differ in their symptoms: (i) the cutaneous form of skin lesions, (ii) the mucocutaneous form, characterized by the destruction of mucous membranes of the nose, mouth, and throat, and (iii) the life-threatening visceral form resulting in systemic infection. The type of leishmaniasis is determined primarily by the species of *Leishmania* causing the infection (2, 4). *Leishmania* RNA viruses (*Leishmaniavirus* spp., LRVs) are common in the New World leishmanias from the subgenus *Viannia*, including *Leishmania guyanensis* and *L. braziliensis*, but are rare in the Old World species (5, 6). The presence of LRV1 in the parasites increases the likelihood of developing mucocutaneous leishmaniasis over the cutaneous form and facilitates the metastatic spread of the parasite (7–10). In addition, LRV1-positive parasites are more resistant to treatments (10). Mice infected with LRV1-positive strains of *L. guyanensis* exhibit greater footpad swelling and higher parasite numbers than mice infected with LRV1-negative strains (9). The evidence that LRV1 contributes to the increased severity of leishmaniasis in humans indicates that eliminating LRV1 from the parasites might alleviate symptoms and facilitate treatment (10). This idea is supported by the observation that mice vaccinated against LRV1 were protected against infection by LRV1-positive *L. guyanensis* (11).

LRVs belong to the family *Totiviridae* of viruses with linear double-stranded RNA (dsRNA) genomes (12). Each LRV1-positive cell contains only 10 to 15 virions of LRV1 (7, 13). Most of our understanding of totivirus biology is based on studies of the L-A virus of Saccharomyces cerevisiae (15, 16). The structure of L-A virus has been characterized by cryo-electron microscopy (cryo-EM) and X-ray crystallography (17, 18). In addition, the structures of Giardia lamblia virus (19), infectious myonecrosis virus (20), Trichomonas vaginalis virus 1 (21), and *Helminthosporium victoriae* virus 190S (22) have been determined to resolutions of 5 to 10 Å using cryo-EM. Totiviruses have nonenveloped, spherical capsids with icosahedral symmetry formed by 120 copies of a capsid protein assembled in asymmetric dimers (23). The atomic-resolution structure of the capsid of L-A virus enabled identification of the mRNA decapping site, which is important for the efficient expression of L-A virus genes, as discussed below (18).

The 5,283-nucleotide-long genome of LRV1 encodes the capsid protein and RNA-dependent RNA polymerase (12, 24). The initiation of translation of the totivirus capsid protein is enabled by an internal ribosomal entry site located at the 5′ end of the virus mRNA (24–26). The translation of the sequence coding the RNA polymerase requires a ribosomal frameshift that enables a ribosome to pass over the stop codon that normally terminates the synthesis of the capsid protein (24–26). A few copies of the capsid protein with RNA polymerase extensions at the C terminus are incorporated into each virion instead of the normal capsid proteins (27). Totiviruses exist as complete virions in the cytoplasm of infected cells (28). Inside the virions, totivirus RNA polymerases synthesize single-stranded copies of the genomes that are extruded from the particles to serve as mRNAs and for the assembly of new virions (17, 23). The double-stranded form of the totivirus RNA genome inside a capsid is protected from degradation by the cytoplasmic Argonaut system (29). In contrast, totivirus mRNAs are not capped at the 5′ end, because they are produced by the viral RNA polymerases (16). Therefore, they may be degraded in the cytoplasm by the cellular RNA exonucleolytic system (30). The maturation of mRNAs of kinetoplastid protists, including *Leishmania* spp., involves *trans*-splicing, in which an identical 39-nucleotide-long mini-exon called the spliced leader is attached to the 5′ end of transcripts (31, 32). Before *trans*-splicing, the mini-exon is capped with a (5′–5′) triphosphate-linked 7-methylguanosine, and the first four nucleotides are modified (33–35). The resulting CAP-4 of *Leishmania* has the structure m7G(5′)ppp(5′)m6(2)AmpAmpCmpm3Ump (33, 35). It has been shown that the capsid proteins of L-A virus possess enzymatic activity to remove the 5′ caps from cellular mRNAs and possibly also transfer them to the viral RNAs (15, 36–40). It has been proposed that the decapped cellular mRNAs serve as decoys that overload the cellular RNA exonucleolytic system and the transferred 5′ caps protect virus mRNAs from degradation (35, 41). However, the decapping or cap-transferring activity of the LRV1 capsid protein has not been experimentally demonstrated.

The decapping or cap-transferring active site of totivirus capsid proteins presents a potential target for small molecule inhibitors, since eukaryotic cells do not possess this enzymatic activity (42). Here, we present the cryo-electron microscopy reconstruction of virus-like particles of LRV1 determined to a resolution of 3.65 Å. The capsid is built from asymmetric dimers of capsid proteins, each of which forms a positively charged cleft. Molecular docking suggested that the clefts might be the decapping sites for leishmania mRNAs.

## RESULTS AND DISCUSSION

### Structure of LRV1 capsid.

**FIG 1:**
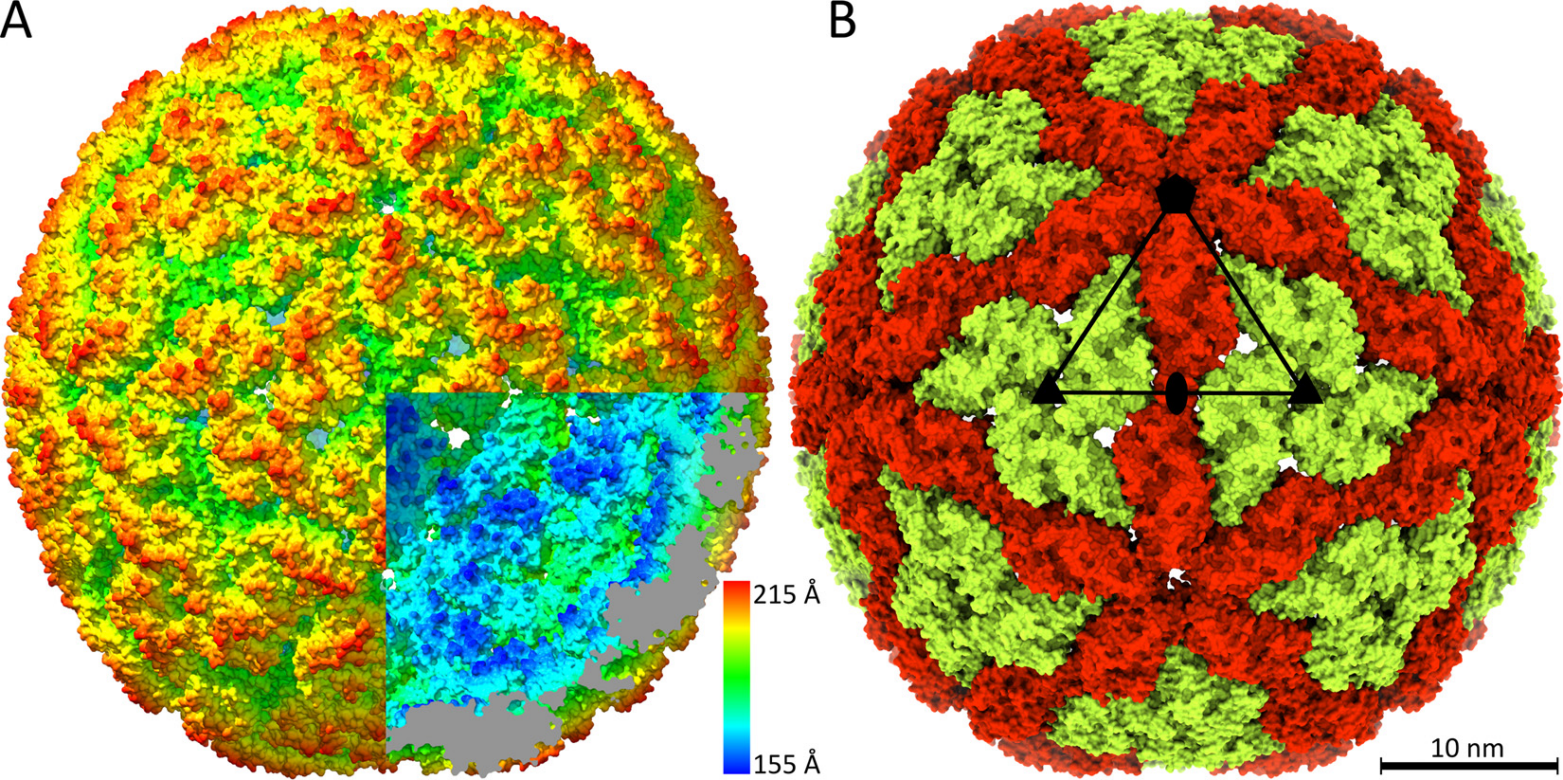
Structure of the LRV1 capsid. (A) Surface representation of cryo-EM reconstruction of the LRV1 capsid, rainbow colored based on distance from particle center. The front bottom right octant of the particle has been removed to show the interior. Clipped density is shown in gray. (B) Organization of the LRV1 capsid, with subunit A shown in red and subunit B shown in green. The borders of one icosahedral asymmetric unit are outlined with a black triangle, with the positions of the 5-fold axis indicated as a pentamer, the 3-fold axes as triangles, and the 2-fold axis as an oval. Scale bar, 10 nm.

The structure of the virus-like particle of LRV1 was determined to a resolution of 3.65 Å using cryo-electron microscopy and single-particle reconstruction (Fig. 1; see Fig. S1 and Table S1 in the supplemental material). The capsid is spherical in shape, with an outer diameter of 424 Å (Fig. 1A). The thickness of the protein shell is 40 Å, and the diameter of the capsid cavity is 344 Å. The particle is built from 120 copies of capsid protein organized with icosahedral symmetry (Fig. 1). The two subunits in the icosahedral asymmetric unit are in nonequivalent binding environments. Subunit A connects 2-fold and 5-fold axes of icosahedral symmetry of the capsid, whereas three B subunits are in contact around the icosahedral 3-fold axes (Fig. 1). Overall, the capsid can be imagined as built from 12 pentons, each composed of five dimers of the capsid protein (Fig. 1B). This arrangement of subunits is a common feature of inner capsid shells of viruses with double-stranded RNA genomes of members of the *Totiviridae*, *Reoviridae*, *Cystoviridae*, *Partitiviridae*, and *Picobirnaviridae* (18, 43–46). It has been speculated that this capsid organization is required for the replication of viral RNA genomes inside of virions by RNA-dependent RNA polymerases, which are expressed as C-terminal extensions of capsid proteins (16). The packaging density of the 5,283-nucleotide-long double-stranded RNA genome in the LRV1 capsid is 5.9 Å^3^/Da, providing twice the space per nucleotide than that available for the double-stranded DNA in the heads of tailed bacteriophages (47–49). The lower RNA density in the LRV1 virion enables the movement of the RNA inside the capsid during its replication.

### Capsid proteins and their asymmetric interactions.

**FIG 2:**
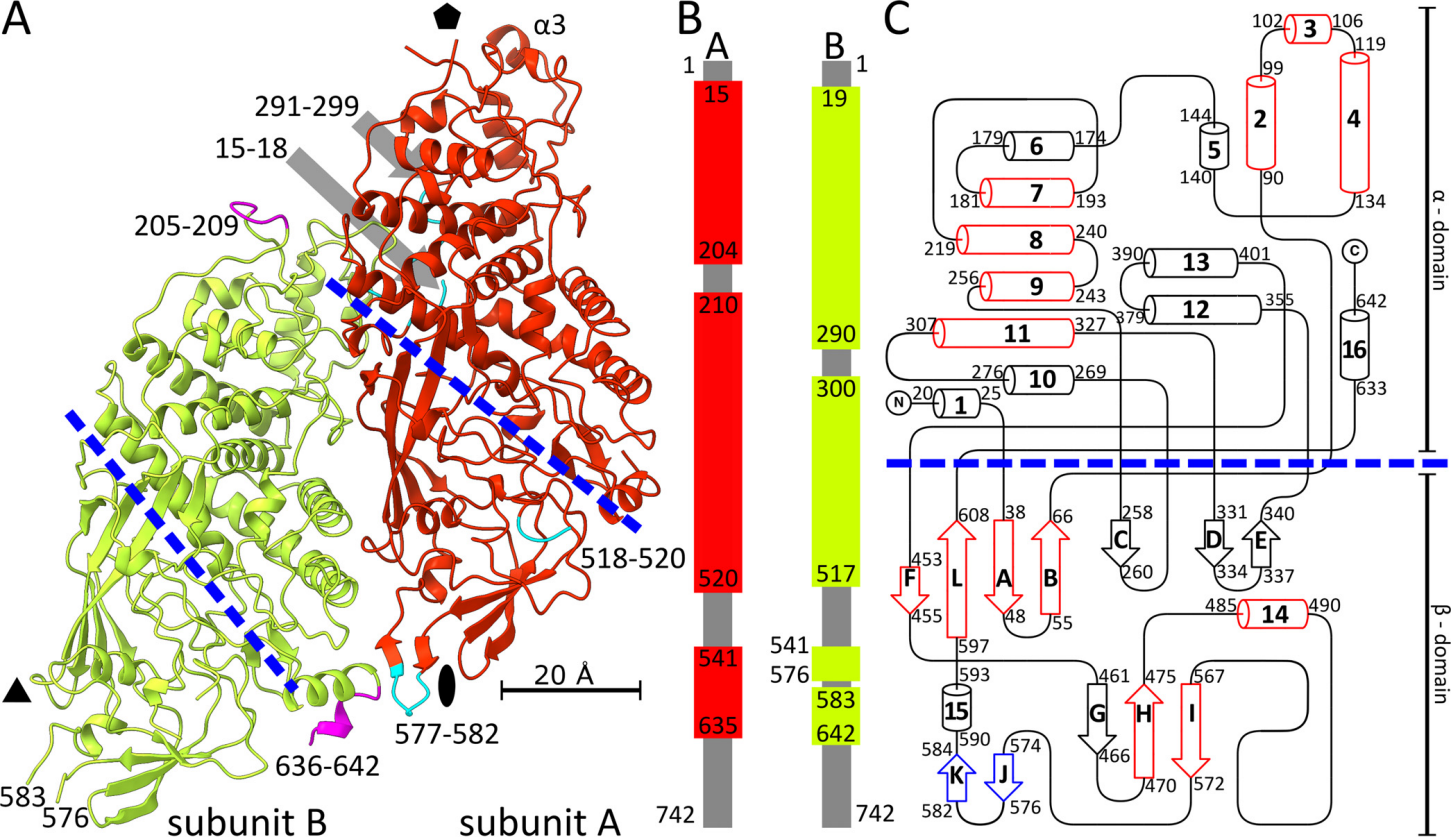
Structure of LRV1 capsid proteins. (A) Cartoon representation of two capsid proteins of LRV1 forming icosahedral asymmetric unit. Subunit A is shown in red, and subunit B in shown in green. Divisions of the subunits into alpha and beta domains are indicated by blue dashed lines. The positions of icosahedral symmetry axes are indicated by an oval for 2-fold, a triangle for 3-fold, and a pentagon for 5-fold. Residues 15 to 18, 291 to 299, 518 to 520, and 577 to 582, which are resolved in subunit A but not in B, are highlighted in cyan. Residues 205 to 209 and 636 to 642, which are resolved in subunit B but not in subunit A, are highlighted in magenta. (B) Scheme showing parts of subunits A (red) and B (green) that are resolved in cryo-EM reconstruction of the LRV1 capsid. Parts of the proteins that were not resolved are shown in gray. (C) Diagram of secondary structure elements of the LRV1 capsid protein. α-Helices are shown as cylinders numbered from 1 to 15, and β-strands are shown as arrows labeled A to L. β-Strands J and K, which are resolved in subunit A but not in subunit B of LRV1, are shown in blue. Secondary structure elements that are shared between LRV1 and L-A virus are highlighted in red.

The two capsid proteins forming the icosahedral asymmetric unit of LRV1 differ in their structures, despite being identical in their amino acid sequences (Fig. 1B and 2A). The structure of subunit A could be built for residues 15 to 204, 210 to 520, and 541 to 635 out of 742, whereas that of subunit B was determined for residues 19 to 290, 300 to 517, 541 to 576, and 583 to 642 (Fig. 2B). Both subunits have the approximate shape of a 100-Å-long cylinder with a diameter of 40 Å. The structures of the two subunits can be superimposed with a root mean square deviation (RMSD) of 0.86 Å for 501 pairs of corresponding Cα atoms out of 586 residues available for the comparison. The two subunits have a similar distribution of secondary structure elements and can be divided into the α-domain, which is formed by α-helices 1 to 13 and 16, and the β-domain, which contains β-strands A-L and α-helices 14 and 15 (Fig. 2C). The core of the α-domain is formed by α-helices 4, 5, 7 to 9, 10, and 12, whereas the shorter helices 2, 3, 6, and 11 are located at the periphery. The β-domain contains antiparallel β-sheets FLABC, GHI, DE, and JK (Fig. 2C).

Structural differences between A and B subunits in the LRV1 capsid, which reflect their nonequivalent interactions with the remainder of the capsid, are located primarily at protein-protein interfaces (Fig. 1B and 2A). The interface between subunits A and B from one icosahedral asymmetric unit includes the interaction of residues 290 to 300 from the loop between helices 10 and 11 of subunit A with residues 158 to 166 of subunit B (Fig. 2A). Residues 290 to 300 from subunit A stabilize residues 15 to 19 of the N terminus within the same subunit. In contrast, residues 290 to 300 and 15 to 18 are not resolved in subunit B. Furthermore, subunits A and B differ in the positions of the loop containing helix 3, formed by residues 101 to 112 (Fig. 2A). The loop of subunit A interacts with another subunit A, related by an icosahedral 5-fold axis. In contrast, residues 101 to 112 of subunit B are exposed to the interior of the capsid and are not involved in protein-protein contacts (Fig. 2A). Residues 573 to 586 forming the JK β-hairpin of subunit A interact with the GH loop of neighboring subunit A related by a 2-fold axis (Fig. 2A). The corresponding loop in subunit B points away from the particle center, and residues 577 to 582 are not resolved in the cryo-EM map (Fig. 2A).

### Interactions between capsid proteins.

**FIG 3:**
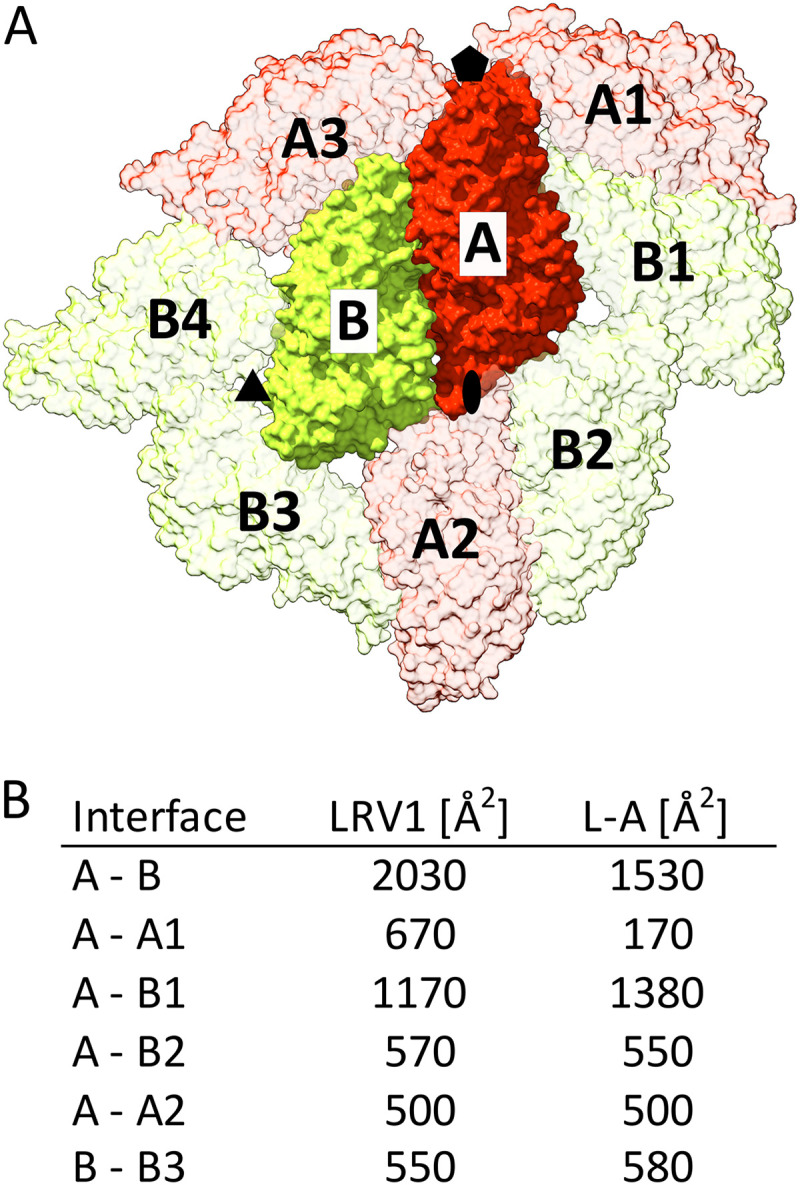
Intersubunit interfaces in the LRV1 capsid. (A) Diagram of interaction interfaces in the LRV1 capsid. Subunits A are shown in red, and subunits B are shown in green. The subunits are numbered to indicate to which icosahedral asymmetric unit they belong. (B) Table of buried surface areas of intersubunit interfaces as indicated in panel A of LRV1 and L-A virus.

The largest interface in the LRV1 capsid between subunits A and B has a buried surface area of 2,000 Å^2^, whereas the remaining interfaces are smaller than 1,200 Å^2^ (Fig. 3). It is likely that the asymmetric dimer of capsid proteins interacting through the largest interface represents the building block from which the LRV1 capsids assemble. The sizes of buried surface areas of interfaces in L-A virus are similar to those of LRV1 and indicate that homologous dimers of L-A proteins may also be the assembly intermediates of this virus (Fig. 3B).

### Pores in the capsid as channels for release of RNA genome and entry of nucleotides.

**FIG 4:**
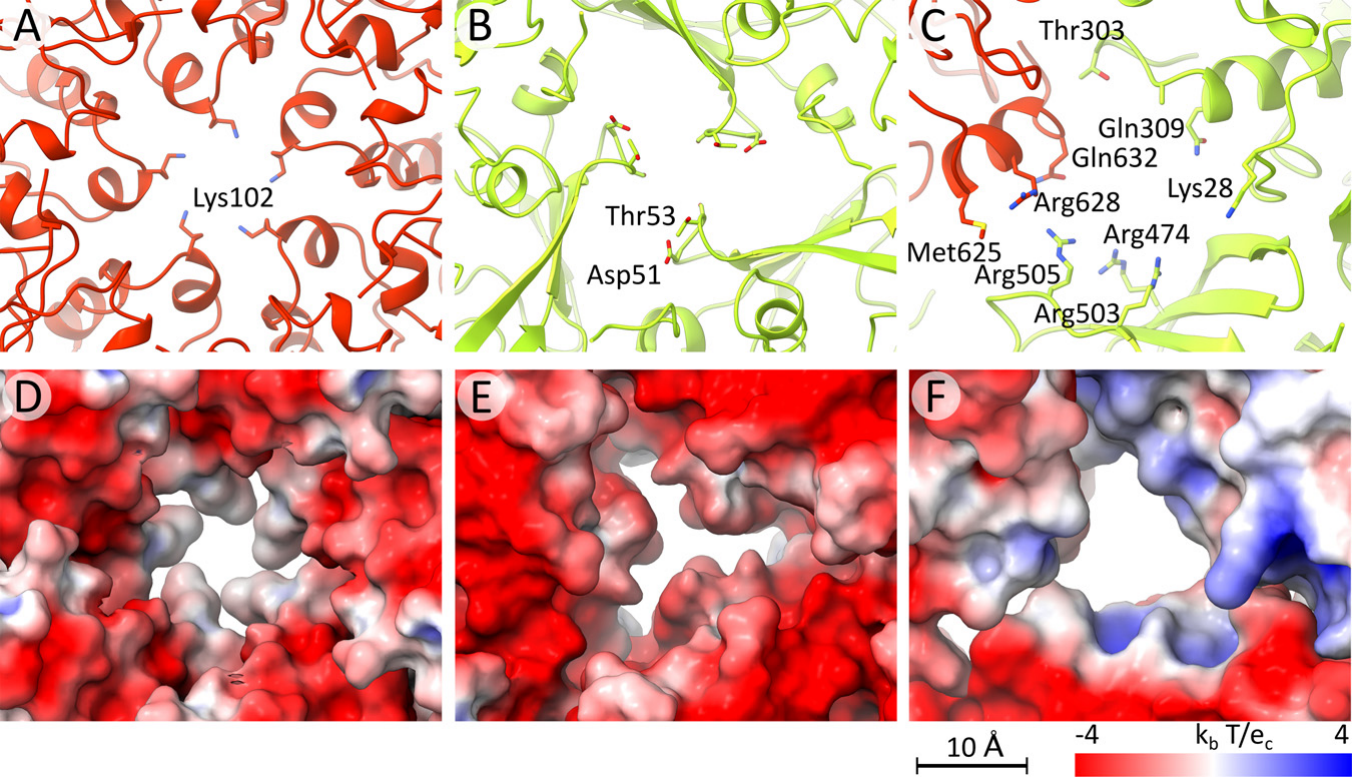
Pores in the LRV1 capsid. (A to C) Cartoon representations of capsid proteins of LRV1 forming pores on 5-fold (A) and 3-fold (B) symmetry axes. An additional pore is formed at the intersubunit interface approximately in the middle between the 3-fold and 2-fold symmetry axes of the capsid (C). Subunits A are shown in red, and subunits B are shown in green. Side chains of residues forming the narrowest constriction in the pore are shown in stick representation. (D to F) Surface representation of pores colored according to charge distribution. Blue indicates positive charge, white is neutral, and red is negative.

To enable the translation and assembly of new virions, nascent RNAs are released from LRV1 particles into the cytoplasm of *Leishmania* cells. Pores along 5-fold symmetry axes of the capsid have been shown to serve as channels for mRNA release from the particles of dsRNA viruses of the *Totiviridae*, *Reoviridae*, *Cystoviridae*, *Partitiviridae*, and *Picobirnaviridae* (18, 43–46, 50). The capsids of LRV1 contain pores with a diameter of 10 Å along the 5-fold axes (Fig. 4A). The pores on 5-fold axes in the LRV1 capsid may be enlarged to a diameter of 13 Å by the movements of side chains of Lys102, which form the narrowest constriction (Fig. 4A). The channels on 5-fold axes of the LRV1 capsid are negatively charged, which may facilitate the exit of the RNA (Fig. 4D). The LRV1 particle contains additional pores with a diameter of 9 Å along 3-fold axes of the capsid and 12-Å-diameter pores in the middle between the 3-fold and 2-fold symmetry axes (Fig. 4B, C, E, and F). The pores along the 5-fold axes, as well as the smaller pores, probably enable the entry of nucleotide triphosphates into the capsid to serve as substrates for RNA synthesis.

### Comparison to the capsid proteins of other dsRNA viruses.

**FIG 5:**
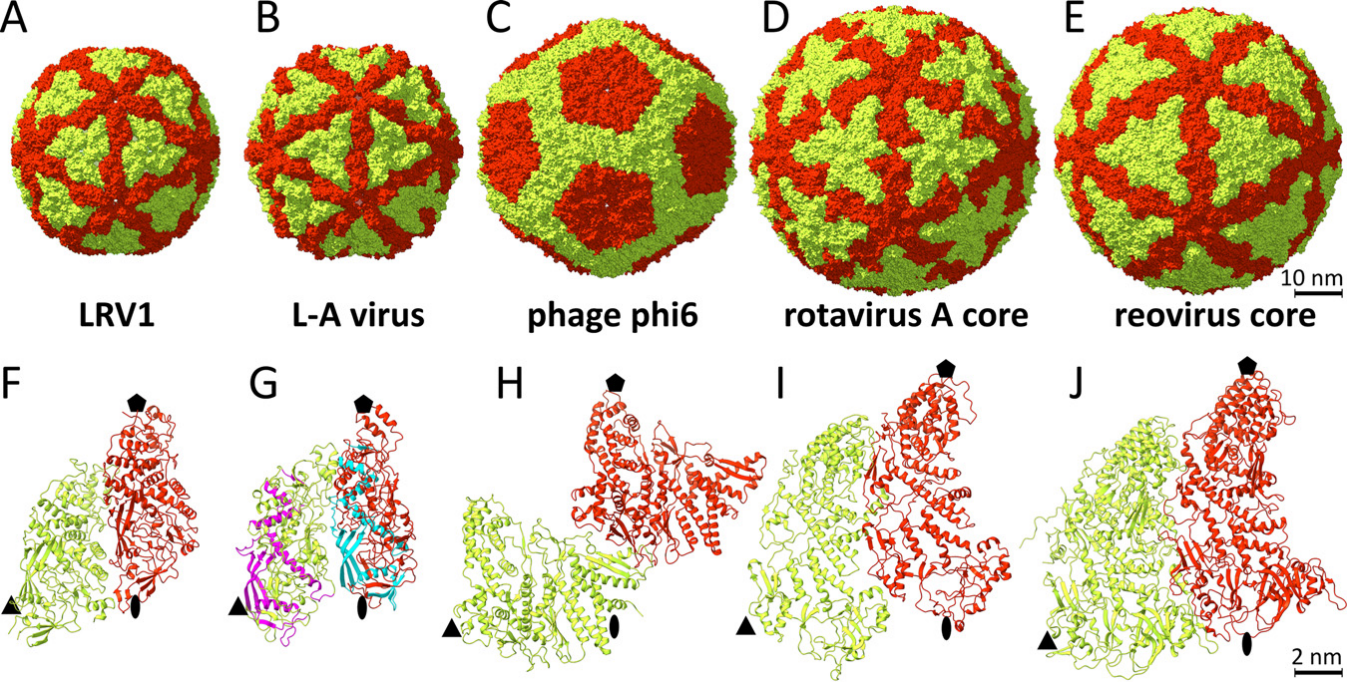
Comparison of capsids and inner capsid shells of viruses that replicate their double-stranded RNA genomes inside particles. (A to E) Surface representations of LRV1 (A), L-A virus (B) (18), phage phi6 (C) (44), inner core of rotavirus A (D) (79), and inner core of reovirus (E) (80). Subunits A are shown in red, and subunits B are shown in green. Scale bar, 10 nm. (F to J) Cartoon representation of proteins forming icosahedral asymmetric units of viruses. Parts of capsid proteins of L-A virus that can be superimposed onto the LRV1 structure are highlighted in cyan and magenta in subunits A and B, respectively (G). Positions of icosahedral symmetry axes are indicated by pentamers for 5-fold, triangles for 3-fold, and ovals for 2-fold axis.

LRV-1, L-A virus, bacteriophage phi6, and inner capsid shells of reovirus and rotavirus A share the general morphology of an icosahedral capsid built from 120 subunits but differ in the structures of their coat proteins (Fig. 5). The structure of LRV1 can be superimposed onto that of L-A virus with an RMSD of 4.5 Å for 423 out of 643 Cα atoms available for the comparison. The two proteins share 11% sequence identity. The capsid proteins of the LRV1 and L-A viruses share α-helices 2, 4, 9, and 11 and β-sheets DE and FLAB (Fig. 5G). The structure of LRV-1 cannot be meaningfully superimposed onto those of phage phi6 and the inner cores of rotavirus A and reovirus. Although the structures of the capsid proteins of dsRNA viruses are different, the functional motifs such as negatively charged pores along the 5-fold axes and positioning of the C terminus of the capsid protein on the inside of the capsid are conserved.

### Putative cleavage sites for 5′ mRNA caps in the LRV1 capsid.

**FIG 6:**
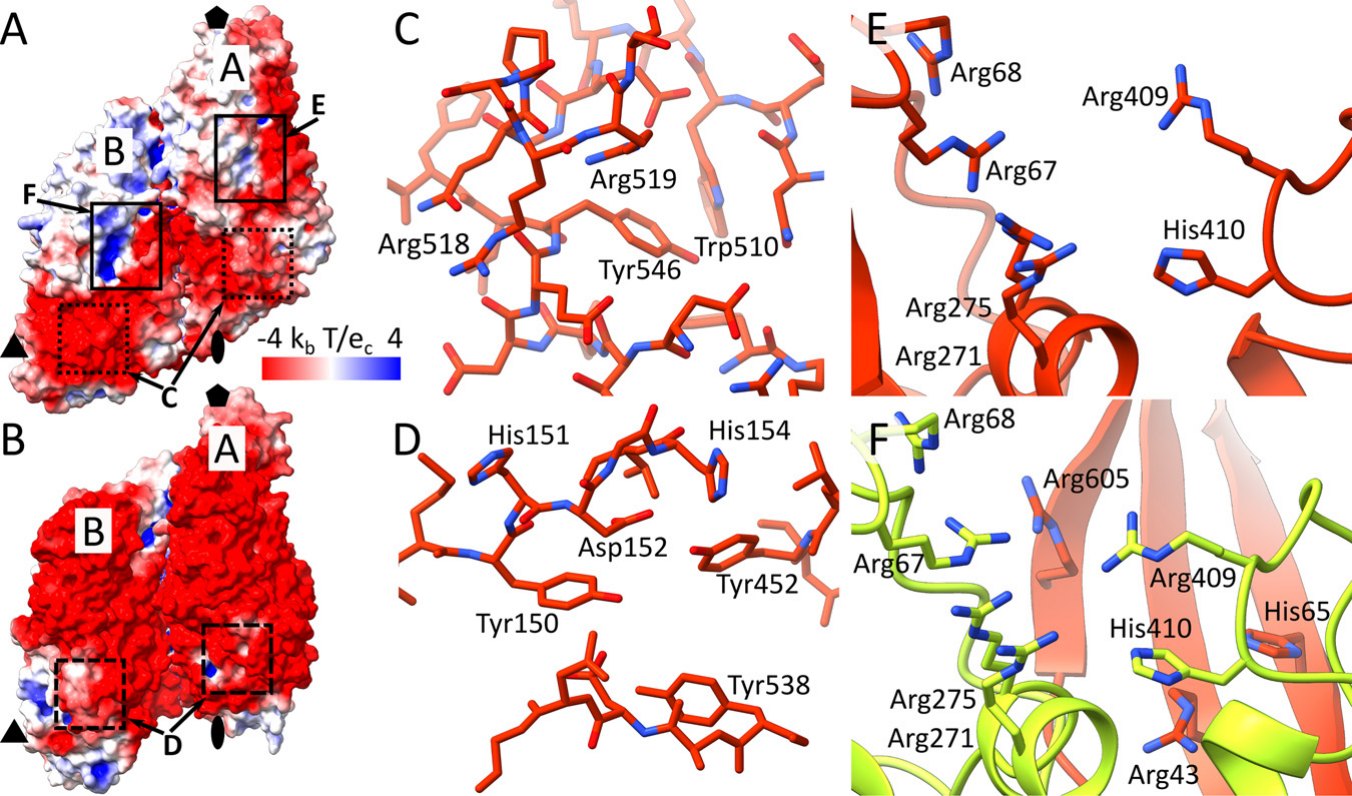
Putative mRNA cap cleavage site of LRV1 is distinct from that of L-A virus. (A and B) Molecular surfaces of icosahedral asymmetric units colored according to charge distribution of LRV1 (A) and L-A virus (B). (A) Putative mRNA binding sites of LRV1 are indicated by black rectangles, and positions corresponding to the decapping sites of L-A virus are indicated by dotted rectangles. (B) Positions of RNA decapping sites in L-A virus are indicated by dashed rectangles. (C and D) Comparison of structures of putative cap cleavage site in L-A virus (D) and the corresponding region in LRV1 (C). (E and F) Details of putative RNA decapping sites in subunits A (E) and B (F) of LRV1. Subunits A are shown in red, and subunits B are shown in green.

*Leishmania* mRNAs have identical 39-residue-long spliced-leader sequences at their 5′ ends, which include an unusual 5′ cap with four modified nucleotides called CAP-4 (33, 51). RNA molecules synthetized by the RNA-dependent RNA polymerase of LRV1 lack CAP-4 and therefore may be degraded by the cytoplasmic exonucleolytic system of *Leishmania* cells. Capsid proteins of totiviruses L-A and L-BC have been shown to cleave off the 5′ caps from host mRNAs, which may overload the exonucleolytic system of cells and thus prevent the virus RNAs from degradation (15, 35, 41). The mRNA decapping may improve the survival of LRV1 RNAs in the cytoplasm of leishmania cells. However, the decapping activity of LRV1 capsid proteins has not been experimentally demonstrated. The mRNA decapping site in L-A virus is formed by Tyr150, His151, Asp152, His154, Tyr452, Tyr538, and Asp540 from the β-domain (Fig. 6A and C) (18). However, the corresponding part of the LRV1 capsid protein has a different structure, and there is no evidence that it is involved in 5′ cap binding and cleavage (Fig. 6B and D). The surface of the LRV1 capsid is negatively charged except for the positively charged cleft in each capsid protein (Fig. 6A). The 12-Å-wide cleft is formed by Arg271, Arg275, Lys65, Arg67, Arg68, Arg409, and His410 (Fig. 6A, E, and F). The cleft of subunit B is extended by Arg43 and Arg605 from the neighboring subunit A (Fig. 6E). As a result, the cleft in subunit B is more positively charged than that in subunit A (Fig. 6F). The abundance of arginine residues in the putative RNA-binding clefts is reminiscent of arginine-rich motifs of proteins that recognize RNA molecules (52).

**FIG 7:**
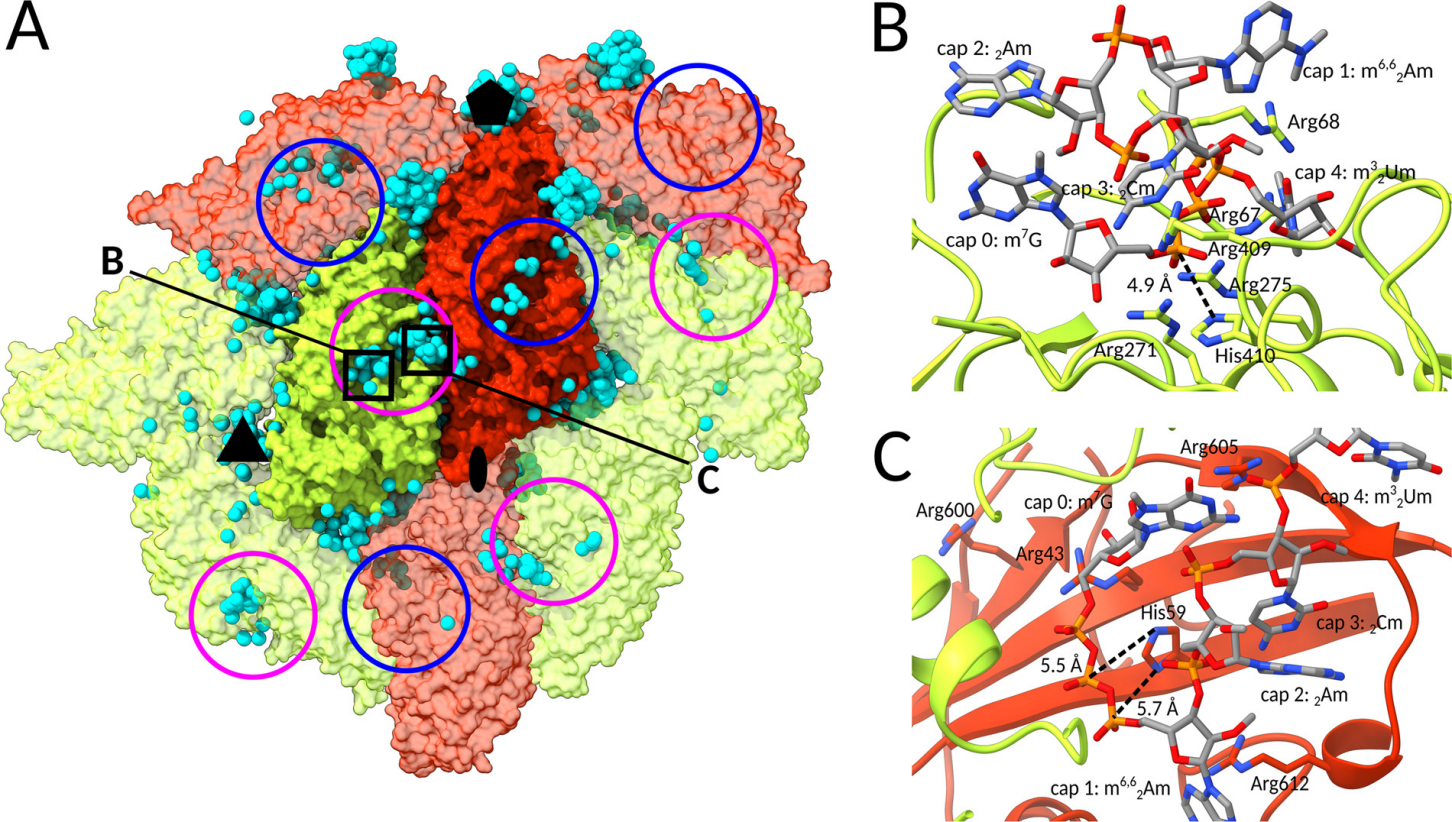
Identification of putative 7-methyl-guanosine cap binding site in the LRV1 capsid. (A) Molecular surface representation of part of the LRV1 capsid. Subunits A are shown in red, and subunits B are shown in green. Cyan spheres indicate positions of the central phosphate in m7Gppp of CAP-4 molecules docked to the structure. The docked molecules cluster in the positively charged clefts of LRV1 capsid proteins and in the pores of the capsid. (B and C) Examples of results from docking of CAP-4 structure that position the m7Gppp of CAP-4 in a position exposed to nucleophilic attack by the amide nitrogen atom from the side chain of His410 (B) or His59 (C).

The cryo-EM reconstruction of LRV1 particles mixed with leishmania mRNAs did not contain density corresponding to CAP-4 (Fig. S2). The cleavage of mRNA by the LRV1 capsid protein may be rapid, and the protein-RNA complex may be present in too few copies to be identified by the classification implemented during cryo-EM reconstruction. Instead, *in silico* docking was performed to characterize the interactions of the LRV1 capsid with CAP-4. Because the structure of leishmania CAP-4 has not been experimentally determined, we used a combination of molecular dynamics and conformational clustering to obtain an ensemble of 28 models representing its thermodynamically relevant structures. Each of the models was docked to the LRV1 capsid. The fitted models with the best docking scores cluster in the positively charged clefts in capsid proteins and in the pores of the capsid (Fig. 7A). The docking of CAP-4 to the pores in the capsid corroborates the proposal that the pores could serve as channels for the entry of nucleotides into the capsid to become substrates for RNA synthesis. The positively charged residues from the clefts electrostatically attract the negatively charged phosphate groups of the RNA (Fig. 7B and C). It has been shown that in L-A virus, the side chain of His145 catalyzes the cleavage of 5′ mRNA caps (18, 36, 53). The docking of CAP-4 to the LRV1 capsid identified complexes in which the triphosphate chain of the 5′ m^7^Gppp cap is in close proximity to the side chains of His59 or His410 (Fig. 7B and C). In these complexes, the phosphorus atoms of the triphosphate chain are exposed to a nucleophilic attack by the amide nitrogen atom from the histidine side chain (Fig. 7B and C).

Our results provide evidence that the mRNA decapping site of LRV1 is not the same as that of L-A virus (Fig. 6A and B). The most likely sites for catalysis of the decapping reaction in LRV1 are the positively charged clefts in α-domains of capsid proteins (Fig. 6A and 7). The differences in the decapping sites of LRV1 and L-A virus indicate divergent evolution (Fig. 6A and B). Nevertheless, the decapping reaction in both LRV1 and L-A virus may be catalyzed by a histidine side chain (Fig. 7B and C). Identification of the putative decapping site provides a target for the development of compounds interfering with the translation of LRV1. Such inhibitors have the potential to become treatments for visceral forms of leishmaniasis caused by LRV1-positive strains of *Leishmania*.

## MATERIALS AND METHODS

### Cloning, expression, and purification of LRV capsid protein.

Total RNA of *Leishmania guyanensis* strain M5313 was isolated using TRI reagent (Sigma-Aldrich) according to the manufacturer’s manual, reverse transcribed into cDNA using a RevertAid first strand cDNA synthesis kit (Thermo Scientific). The *gag* gene (GenBank accession no. MK825886) of LRV1 major capsid protein was PCR amplified using the primers GAGTCTCATATGGCTGATATACCAAACTCTGATAAG and TTTGGAAGATCTGGAGCGCAGCCTCCGTTGTCACCGG. The amplified fragment was inserted into the plasmid pET42b using the restriction sites NdeI and XhoI. The insertion resulted in the addition of a C-terminal 8×His tag to the protein. The construct was expressed in Escherichia coli strain BL21(DE3) in TB medium (12 g/liter tryptone, 24 g/liter yeast extract, 0.4% glycerol, 1% glucose, 170 mM KH_2_PO_4_, 720 mM K_2_HPO_4_). Protein expression was induced by the addition of IPTG (isopropyl-β-d-thiogalactopyranoside) to a final concentration of 0.3 mM to a culture with an optical density at 600 nm (OD_600_) of 0.5. After induction, the culture was allowed to grow for 5 h at 30°C and with shaking at 250 rpm. The resulting culture was centrifuged at 5,000 × *g*, 4°C, for 15 min. Mild sonication (30% power, 1-min process time with 10-s burst/10-s pause; Qsonica Q700 instrument) was used to resuspend pelleted cells in lysis buffer (300 mM NaCl, 50 mM Tris [pH 8], 1 mM EDTA, 5 mM β-mercaptoethanol, 0.1% Triton X-100, 1 mM MgCl_2_, 10 μg/ml DNase I, 1 μg/ml lysozyme, 20 mM imidazole). The cell suspension was lysed by three rounds of mechanical disruption with Emulsiflex C3 (Avestin). The lysate was cleared by centrifugation at 10,000 × *g* for 20 min at 4°C and filtered through a syringe filter with 0.45-μm pores (Millipore). The LRV1 particles were purified using a 5-ml HisTrap column (GE Healthcare). The particles were eluted using a 400 mM imidazole solution with 300 mM NaCl, 50 mM Tris [pH 8], 5 mM β-mercaptoethanol, and 0.1% Triton X-100. Fractions containing the LRV1 capsids were pooled, concentrated, and further purified in a Superdex 26/200 pg column (GE Healthcare). Size exclusion chromatography was utilized to transfer the protein into phosphate buffer (20 mM sodium phosphate [pH 7.4], 300 mM NaCl, 5 mM β-mercaptoethanol). Fractions containing the purified LRV1 capsids were pooled and concentrated to 0.16 mg/ml.

### Cryo-electron microscopy and single-particle reconstruction.

The virus-like particles of LRV1 at a concentration of 0.16 mg/ml (4 µl) were applied to holey carbon grids (Cu, 300 mesh, R2/1; Quantifoil micro tools) and blotted and plunge frozen in liquid ethane using a Vitrobot Mark IV (Thermo Fisher Scientific) with the following settings: blot time, 2 s; wait time, 10 s; and blot force, 0. Data were collected using a Titan Krios (Thermo Fisher Scientific) electron microscope operated at 300 kV and aligned for parallel illumination in nanoprobe mode. Individual images were recorded with an FEI Falcon II direct electron detection camera under low-dose conditions (17 e^−^/Å^2^) with underfocus values ranging from 0.5 to 3 µm at a magnification of ×75,000, resulting in a pixel size of 1.063 Å/px. Each image was recorded in movie mode with a 0.38-s acquisition time and saved as seven separate movie frames. The frames from each exposure were aligned to compensate for drift and beam-induced motion during image acquisition using the program MotionCor2 (54). A total of 25,849 particles were manually selected using e2boxert.py from the software package EMAN2 (55). Contrast transfer function parameters for each micrograph were automatically estimated using the program CTFFIND4 (56). The LRV1 particle and LRV1 particle in solution with added leishmanial RNA were reconstructed using the package RELION 2 (57). The initial model was generated from the structure of the L-A virus of yeast (PDB ID 1M1C), which was low-pass filtered to 40 Å. The data sets were subjected to multiple rounds of two-dimensional (2D) and 3D classifications, and the final reconstruction was performed using the program 3dautorefine. The resulting unfiltered electron density maps were masked with threshold masks, which were created using the RELION mask_create function. The occurrence of overmasking was monitored by inspecting the shape of the Fourier shell correlation (FSC) curve. Furthermore, the shapes of the FSC curves of phase-randomized half-data sets with the applied mask were checked. The resolution of the final reconstruction was estimated as the value at which the FSC curves fell to 0.143 in accordance with the “gold standard” procedure for resolution determination (58). The reconstruction of the LRV1 particle from the sample containing leishmania RNA did not contain any density that could be attributed to the RNA bound to the particles. Therefore, all images of particles were combined for the reconstruction of the native LRV1 virus-like particle.

### All-atom simulation of CAP-4.

The RNA cap simulated in this work reproduces the 5′ mRNA CAP-4 structure from *Leishmania* (59). A single-stranded RNA model containing standard nucleotides was generated using the make-na server (http://wiki.christophchamp.com/index.php?title=Make-na). The posttranscriptional modifications and the 7-methyl-guanosine-5′-diphosphate cap (m7Gpp) were introduced manually using the program PyMol (Shrödinger, LLC; The PyMOL Molecular Graphics system, version 2.2, 2018).

The RNA cap was solvated with the TIP3P water model (60) in a cubic box with a size of 4.4 nm. NaCl ions were added to a concentration of 150 mM with excess ions to neutralize the system net charge. Periodic boundary conditions were applied in all directions. The system was energy minimized first with and then without nonhydrogen atoms restrained to initial positions. The steepest descent algorithm with convergence criteria of 500 kJ/mol/nm was applied. The system was subsequently equilibrated for approximately 200 ns with position restraints that were gradually relaxed.

To increase the configurational sampling and obtain relevant structures for docking, an enhanced sampling of the CAP-4 conformational space was achieved via a 200-ns solute-tempering replica exchange simulation (61). We used six replicas with effective temperatures of the solute at 300, 325, 353, 383, 415, and 450 K. All the replicas started from the equilibrated structure, and the exchange of configurations between neighboring replicas was attempted every 500 ps with an acceptance ratio of about 25%. Each replica simulation was conducted in an isothermal-isobaric ensemble with constant particle number, pressure of 10^5^ Pa, temperature of 300 K, and integration time step of 2 fs. Pressure and temperature were controlled using a Parrinello-Rahman barostat (62) with a coupling time of 5.0 ps and a velocity-rescaling thermostat (63) with a coupling time of 0.5 ps, respectively. The geometry of water was kept rigid using the SETTLE algorithm (64). All nonwater covalent bonds with hydrogen atoms were constrained by the LINCS algorithm (65). Long-range electrostatic interactions were treated using the particle mesh Ewald method (66) using a Fourier spacing of 0.12 nm, real space cutoff of 1.2 nm, and particle mesh Ewald order of 4. Short-range Lennard-Jones interactions were truncated at 1.2 nm with the force smoothly switched to zero at the cutoff distance. The neighbor list was updated every 0.04 ps with a cutoff distance of 1.2 nm. Long-range correction was not used for the dispersion interactions. All atomistic simulations were carried out using the Molecular Dynamics package GROMACS version 5.1.4 (67) combined with PLUMED version 2.3 (68), and the force field parameters were based on a CHARMM36 port for GROMACS (69–73) customized to account for CAP-4 connectivity and partial atomic charges.

### Cluster analysis.

In order to obtain a pool of representative CAP-4 structures, the conformations from the simulation of the lowest replica were clustered based on RMSD (root mean square deviation) of distances of corresponding nonhydrogen atoms. The analysis was performed employing the gmx cluster tool from GROMACS with the gromos method (74) and a cutoff of 0.15 nm. Only the clusters containing at least 100 out of the 20,000 available configurations were retained. The representative CAP-4 configuration (i.e., the configuration with the smallest average RMSD from all other configurations in the cluster) was determined for each selected cluster and used for docking.

### RNA cap docking and data analysis.

The representative CAP-4 structures were docked to the LRV1 capsid using the HDOCK server, which was chosen because of its good performance in the recent CAPRI assessments (75–78). To improve the sampling, each of the representative structures was docked three times, each time with a different initial orientation obtained by 90° rotations. For each protein–CAP-4 pair, the top scoring 100 poses were retained for further analyses, yielding a total of 11,200 poses, which were sorted by the best score.

### Data availability.

Cryo-EM electron density maps have been deposited in the Electron Microscopy Data Bank (https://www.ebi.ac.uk/pdbe/emdb/) under accession numbers EMD 10722 and EMD 10745, and the fitted coordinates have been deposited in the Protein Data Bank under PDB ID 6Y83. The sequence of *Leishmania* RNA virus 1 isolate LRV1-4 major capsid protein mRNA is available in GenBank under accession number MK825886.

## Supplementary Material

Supplemental file 1
