# Supplementary material for: Capsid Structure of Leishmania RNA Virus 1
Source: J Virol. 2021 Jan 13;95(3):e01957-20. doi: 10.1128/JVI.01957-20 (PMC7925086; doi:10.1128/JVI.01957-20)
Supplement: Supplemental file 1 [file JVI.01957-20-s0001.pdf]

Supplementary Material
for
Capsid structure of *Leishmania* RNA virus 1

Short title: Capsid structure of *Leishmania* RNA virus 1

Michaela Procházková[1], Tibor Füzik [1], Danyil Grybchuk[1], Francesco Falginella[2], Lucie Podešvová[3], Vyacheslav Yurchenko[3,4], Robert Vácha[1,2], Pavel Plevka[1]

1 - Central European Institute of Technology, Masaryk University, Kamenice 753/5, Brno, 625 00, Czech Republic
2 - Faculty of Science, Masaryk University, Kamenice 753/5, Brno, 625 00, Czech Republic
3 - Life Science Research Centre, Faculty of Science, University of Ostrava, Chittussiho 10, Ostrava, 710 00, Czech Republic
4 - Martsinovsky Institute of Medical Parasitology, Tropical and Vector Borne Diseases, Sechenov University, Malaya Pirogovskaya 20, Moscow, 119435, Russia

Corresponding author: pavel.plevka@ceitec.muni.cz

Table S1. Cryo-EM data and structure quality statistics

Supplementary table 1

Structure	LRV1	LRV1+mRNA
EMDB	EMD-10722	EMD-10745
Magnification	75 000 x	75 000 x
Pixel size (Å)	1.063	1.063
Frames per exposure	7	7
Voltage	300 kV	300 kV
Electron dose (e^-/Å^2)	21	21
Symmetry	icosahedral	icosahedral
Initial particles (no.)	25 849	16 715
Particles used for reconstruction (no.)	16 901	9 932
Map resolution (Å)	3.65	3.76
FSC treshold	0.143	0.143
PDB ID	6Y83	
R factor[+]	0.371	
No. of atoms[§]	9294	
RMSD bond lengths	0.005	
Bad bonds (%)*	0	
RMSD bond angles	0.885	
Bad angles (%)*	0.05	
Ramachandran favored (%)*	96.32	
Ramachandran allowed (%)*	3.68	
Ramachandran outliers (%)*	0	
Poor rotamers (%)*	0.2	
Clashscore (percentile)*	9.07 (97)	
MolProbity score (percentile)*	1.73 (100)	
Cβ deviations (%)*	0	

[+] calculated by phenix.refine for a pentamer of asymmetric units in particle map
[§] number of non-H atoms in asymmetric unit
* values according to Molprobity

Supplementary figures:

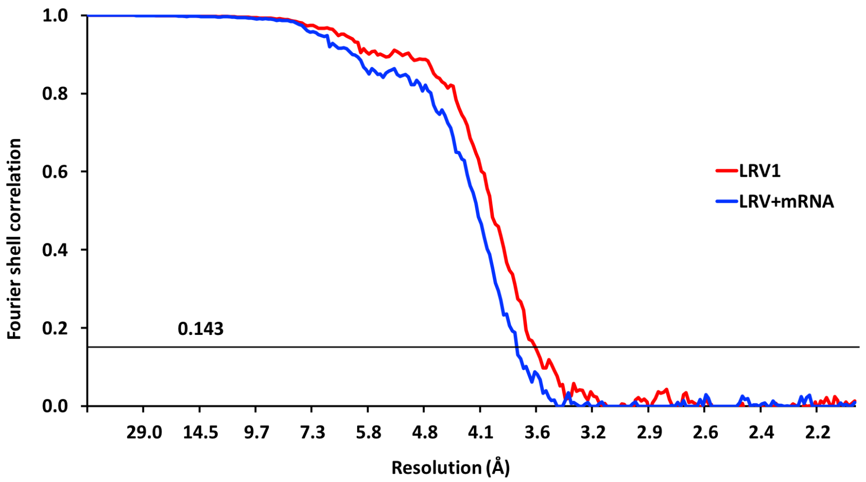

Fig. S1. FSC curves for reconstructions of LRV1 (red) and LRV1 in complex with *Leishmania* mRNA (blue).

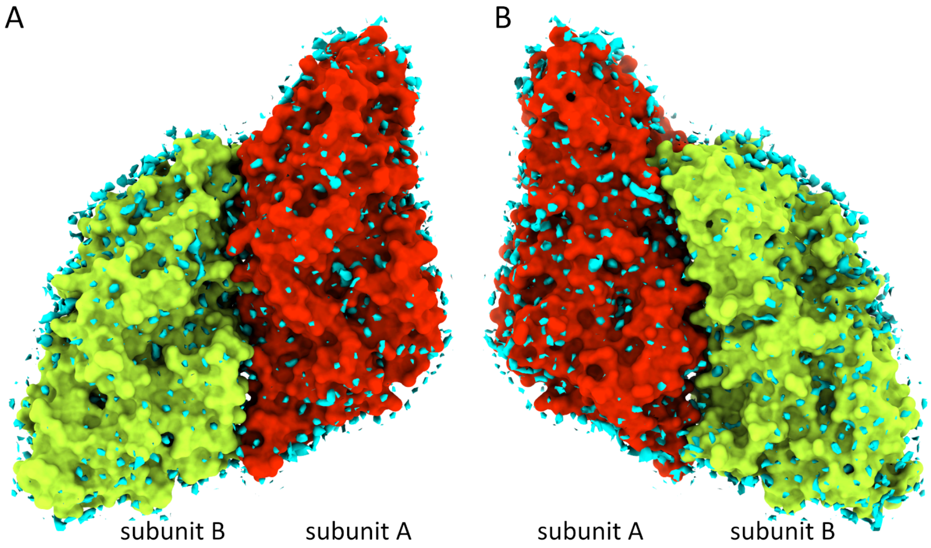

Fig. S2. Reconstruction of LRV1 in complex with leishmania mRNA does not contain density corresponding to mRNA bound to capsid. (AB) Surface representation of icosahedral asymmetric unit of LRV1. Subunit A is shown in red, subunit B in green. View from the outside of a particle (A) and inside (B). Difference map calculated by subtracting Cryo-EM reconstruction of virus-like particle of LRV1 from that of LRV1 virus-like particle mixed with leishmania mRNA is shown in blue at 0.1 s and contains only noise.
